# Engraftment and persistence of *HBB*-base-edited hematopoietic stem cells in nonhuman primates

**DOI:** 10.1126/scitranslmed.adn2601

**Published:** 2025-08-13

**Authors:** Stefan Radtke, Emily Fields, Kyle Swing, Greta Kanestrom, Jonathan S. Yen, Dnyanada Pande, Mark R. Enstrom, Olivier Humbert, Mitchell J. Weiss, David R. Liu, Gregory A. Newby, Hans-Peter Kiem

**Affiliations:** 1 Stem Cell and Gene Therapy Program, Translational Science and Therapeutics, Fred Hutchinson Cancer Center, Seattle, WA, 98109, USA.; 2 Division of Hematology and Oncology, University of Washington School of Medicine, Seattle, WA, 98195, USA.; 3 Department of Hematology, St. Jude Children’s Research Hospital, Memphis, TN, 38105, USA; 4 Merkin Institute of Transformative Technologies in Healthcare, Broad Institute of Harvard and MIT, Cambridge, MA, 02142, USA.; 5 Department of Chemistry and Chemical Biology, Harvard University, Cambridge, MA, 02138, USA; 6 Howard Hughes Medical Institute, Cambridge, MA, 02142, USA.; 7 Department of Genetic Medicine, Johns Hopkins University School of Medicine, Baltimore, MD, 21205, USA.

## Abstract

Sickle cell disease (SCD) is caused by a single nucleotide change in the β-globin chain that adenine base editors can convert into the non-pathogenic Makassar β-globin variant. Here, we evaluated the long-term efficiency and off-target editing potential of autologous Makassar base editing in three rhesus macaques as a step toward human translation. Base editing of CD34^+^CD90^+^ hematopoietic stem cells (HSCs) at the Makassar locus reached greater than 60% efficiency using a bystander nucleotide as a proxy for the sickle cell target in cells from healthy macaques. No impact on myeloid and erythroid colony formation was seen, and clonal analysis revealed that >90% were edited, >20% with biallelic editing. After transplantation of autologous gene-edited HSCs, all three macaques rapidly recovered neutrophils, red blood cells, and platelets with stable editing of 25.6% on average observed across nucleated blood cells. Similarly, the bone marrow stem cell compartment maintained over 20% of cells harboring mono- or biallelic edits. Off-target editing was assessed at over 900 candidate sites, with editing observed at 8 sites, but no selection for or impact of these edits was observed throughout engraftment. These data support further translation of base editing of autologous HSCs for the treatment of patients with SCD.

## One Sentence Summary

Ex vivo beta-globin base-edited hematopoietic stem cells retain multilineage long-term engraftment potential upon autologus transplantation in NHPs.

## INTRODUCTION

Base editing enables the targeted exchange of nucleotides in the genome to correct mutations associated with a wide variety of diseases and malignancies. Multiple ex vivo and in vivo studies in mice have shown that base editors (BEs) can efficiently correct hepatocytes, muscle, retinal, ear, and hematopoietic cells (1). Furthermore, efficient ex vivo modification and/or correction of human hematopoietic stem cells (HSCs) and T cells followed by successful transplantation of gene-modified cells into immunodeficient mice and acute lymphocytic leukemia (ALL) patients, respectively, has been demonstrated (2–10). To further ensure the well-being of patients, rigorous evaluation of the long-term efficiency and safety of this treatment are warranted.

Sickle cell disease is the most common deadly genetic disease worldwide and is caused by a single nucleotide change in the hemoglobin subunit beta gene (*HBB),* which leads to polymerization of hemoglobin (11). Individuals affected by sickle cell disease carry at least one copy of the Glu6Val amino acid substitution, caused by an A→T transversion. Although base editing cannot efficiently correct this transversion back to the wild type state, we and others have shown that adenine base editors can modify the disease-causing nucleotide through a T→C edit leading to a Val6Ala coding sequence (12–14) which is a rare but naturally occurring and benign genotype called the Makassar variant (12, 15, 16). We have previously used the adenine base editor ABE8e, evolved for high activity (17), together with the NRCH Cas9 targeting moiety, developed for an expanded scope of compatible PAM sequences (18), to achieve 68% editing of the sickle globin sequence to Makassar globin sequence in bone marrow repopulating human hematopoietic stem cells that engrafted into immunocompromised mice (12). This demonstrated proof-of-principle that a base editing treatment could directly eliminate the causative sickle cell disease variant as a therapeutic strategy, which could have advantages over approaches that leave the polymerizing sickle cell allele intact.

Although mice are crucial for initial studies to test the toxicity of these new agents and above described treatment strategies, differences in physiology, size, and lifespan, as well as the lack of a fully functional immune system, do not allow for the long-term assessment of potential side effects, impact on the maturation or quantitative representation of all blood lineages, or development of secondary malignancies. The rhesus macaque (*Macaca mulatta*) shares a close evolutionary relationship with humans, demonstrates cross-reactivity for human reagents, and allows for the assessment and testing of aspects of safety, including off-target edits that cause clonal outgrowth or secondary malignancies. Consequently, we evaluated the feasibility and long-term efficiency of autologous ex vivo HSC base editing in this preclinical nonhuman primate (NHP) large animal model using our previously established editing strategy in the β-globin chain to treat sickle cell disease (SCD).

## RESULTS

### Efficient ex vivo base editing of nonhuman primate CD34^+^CD90^+^ HSCs

**Fig. 1.**
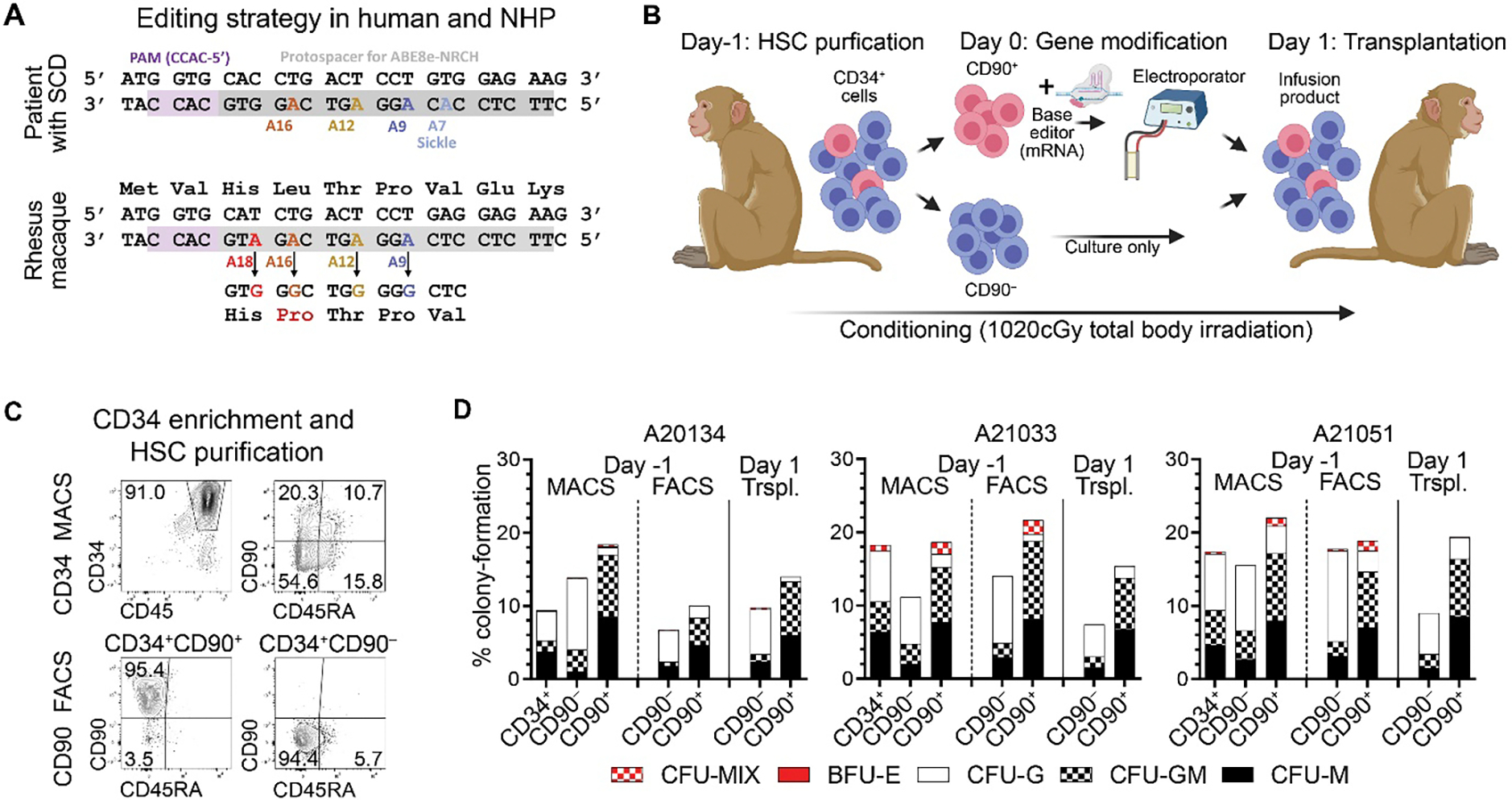
Purification of NHP CD34^+^CD90^+^ HSCs for ex vivo base editing. (**A**) Comparison of the protospacer design for patients with SCD and NHPs. On- and off-target adenines are color coded. (**B**) Experimental outline of autologous ex vivo HSC base editing in the NHP. Briefly, NHPs were pre-treated with G-CSF/SCF for 5 days, BM harvested, CD34^+^ cells enriched via MACS, and CD90^+/−^ FACS purified (Day −1). After overnight stimulation of cells in culture, CD90^+^ HSCs were electroporated with base editor mRNA and sgRNA (Day 0). After over-night resting, edited CD90^+^ HSCs were combined with unedited CD90^−^ cells, and infused into conditioned animals (Day 1). Conditioning was carried out on Day 0 and 1 by giving 4 fractions of 255cGy TBI. (Created in BioRender. Radtke, S. (2025) https://BioRender.com/wmu0r89) (**C**) Representative flow-cytometry of bulk CD34^+^ cells after MACS (top row), and FACS-purified CD34^+^CD90^+^ HSCs (bottom left) as well as CD34^+^CD90^–^ progenitors (bottom right). (**D**) Percentage colony formation (number of colonies / number of seeded cells) of unmodified HSPC subsets after MACS/FACS (Day −1) and of base-edited HSPCs at time of infusion (Day 1) for each NHP. Abbreviations: CFU = colony-forming unit; G = granulocyte; M = monocyte/macrophage; BFU = burst forming unit; E = erythrocyte; MIX = myeloid + erythroid.

SCD is caused by a single nucleotide change (position A7 in the protospacer, Fig. 1A) in the *HBB* (hemoglobin subunit β) gene that can be converted into the non-pathogenic Makassar β-globin (HBB^G^) variant using adenine base editors (ABEs). Because NHPs do not carry the sickle mutation, synonymous bystander editing at position A9, which was previously observed to have nearly identical editing efficiency (12), was monitored as a surrogate for successful gene editing.

To model the efficiency of base editing in wild type NHP CD34^+^CD90^+^ HSCs, a previously designed guide RNA (gRNA) used to target ABE8e-NRCH to human *HBB*^*S*^ (12, 17, 18) was modified in two positions: at position 7 where the causative sickle cell variant would fall in a patient, and at position 18, which is polymorphic between rhesus macaque and human (Fig. 1A, table S1). Three juvenile NHPs were enrolled in this study and ex vivo autologous gene editing performed using our previously reported HSC purification protocol (19, 20) (Fig. 1B, table S1). Briefly, G-CSF (granulocyte colony-stimulating factor)/SCF (stem cell factor)-mobilized CD34^+^ HSPCs (hematopoietic stem and progenitors) were enriched using magnetic beads and the HSC-enriched CD34^+^CD90^+^ as well as the progenitor-enriched CD34^+^CD90^–^ subsets were FACS (fluorescent assisted cell sorting)-purified (Day −1 relative to time of base editing) (Fig. 1C). Purified CD34 subsets were cultured overnight and only CD34^+^CD90^+^ HSCs were base-edited (Day0), whereas unmodified CD34^+^CD90^–^ progenitors were kept in culture. Base-edited CD34^+^CD90^+^ cells rested overnight were mixed with unmodified CD34^+^CD90^–^ cells on Day 1, and infused into total body-irradiated (TBI, 1020cGy) animals.

To ensure maintenance of phenotypical and functional properties of HSPCs for each animal throughout the entire process of ex vivo gene editing, bulk HSPCs were flow-cytometrically assessed and phenotypic subsets introduced into colony-forming cell (CFC) assays using FACS (Fig. 1D). CD34^+^ HSPCs and CD34^+^CD90^+/–^ were analyzed after MACS (magnetic activated cell sorting, Day-1), FACS-purification (Day-1), and at time of infusion (Day1). No impact on the erythro-myeloid differentiation potential was seen throughout the purification and base editing process. In addition, the total number of phenotypical CD34^+^CD90^+^ and CD34^+^CD90^–^ cells at the time of transplant was quantified and the cell dose per kg bodyweight calculated (table S1). All three animals received about 9 to 10 × 10^6^ total CD34^+^ HSPCs per kg body weight with most cells being unmodified CD34^+^CD90^–^ progenitors. The greatest number of CD34^+^CD90^+^ HSCs per kg body weight was administered into A20134 with 1.34×10^6^ CD34^+^CD90^+^ HSCs/kg followed by A20151 receiving 8.73×10^5^ CD34^+^CD90^+^ HSCs/kg and A21033 transplanted with 2.03×10^5^ CD34^+^CD90^+^ HSC/kg, all three above the previously determined minimum of 1.12×10^5^ CD34^+^CD90^+^ HSCs/kg required for hematopoietic recovery (19).

**Fig. 2.**
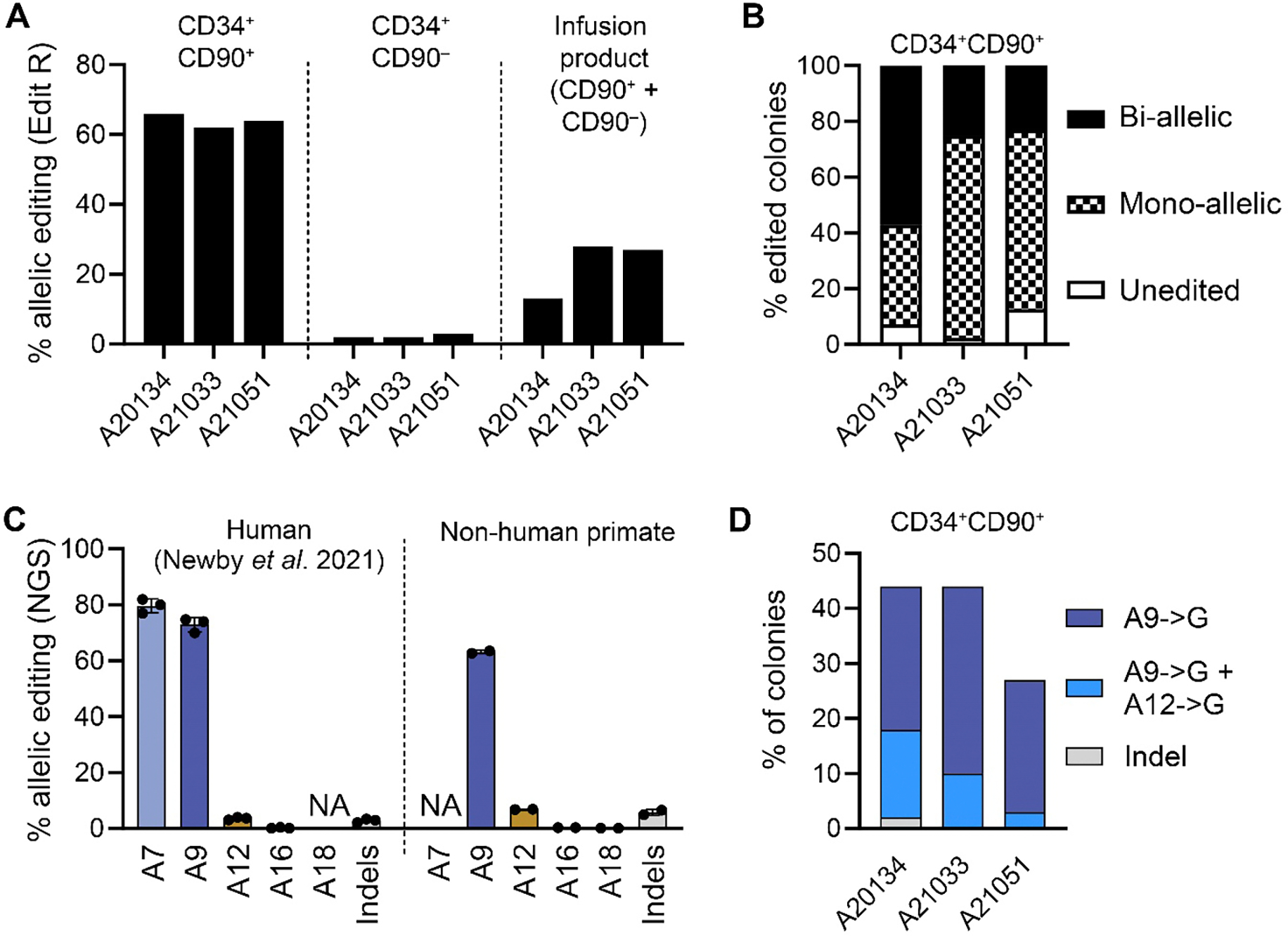
Base editing of the *HBB* gene in NHP HSCs. (**A**) Allelic base editing efficiency in CD34^+^CD90^+^ HSCs, CD34^+^CD90^−^ progenitors, and the combined infusion product determined by EditR analysis of Sanger sequencing at position A9. (**B**) Assessment of mono- and biallelic editing on single colonies from Fig. 1D by Sanger sequencing (EditR) at position A9. (**C**) Comparison of the frequency of allelic editing at all adenines and indels in the protospacer between previously published data from human cells after ex vivo base editing (12) and NHP CD34^+^ HSPCs after ex vivo base editing as assessed by NGS. (**D**) Assessment of editing patterns on a single cell level from colonies in Fig. 1D by EditR analysis of Sanger sequencing.

Base editing in NHP HSPCs was monitored at position A9, a silent bystander mutation that was efficiently introduced in cells of patients with SCD at similar frequencies as the Makassar edit at position A7 (12). At Day1, Sanger sequencing of the *HBB* locus in CD34^+^CD90^+^ HSCs showed greater than 60% editing in all three animals (Fig. 2A). To determine the frequency of mono- and biallelic edits of *HBB*, colonies derived from base edited CD34^+^CD90^+^ HSCs at the day of infusion (Day1) were isolated for clonal analysis (Fig. 2B). In contrast to the bulk analysis (Fig. 2A), more than 90% of colonies demonstrated successful editing at position A9, with most cells edited at one allele and greater than 20% of colonies showing biallelic editing. Lastly, CD34^+^CD90^+^ HSCs were analyzed by next generation sequencing (NGS) to quantify bystander editing at positions A16 and A18 (Fig. 2C). Side-by-side comparison of editing at the different adenines between human and NHP HSCs were closely matching, confirming specific activity of the ABE in the desired editing window. Of note, clonal analysis revealed that the silent bystander edit at position A12 was only found to occur in cells also edited at position A9 (Fig. 2D).

### Rapid recovery and multilineage engraftment in the peripheral blood and bone marrow after base editing

**Fig. 3.**
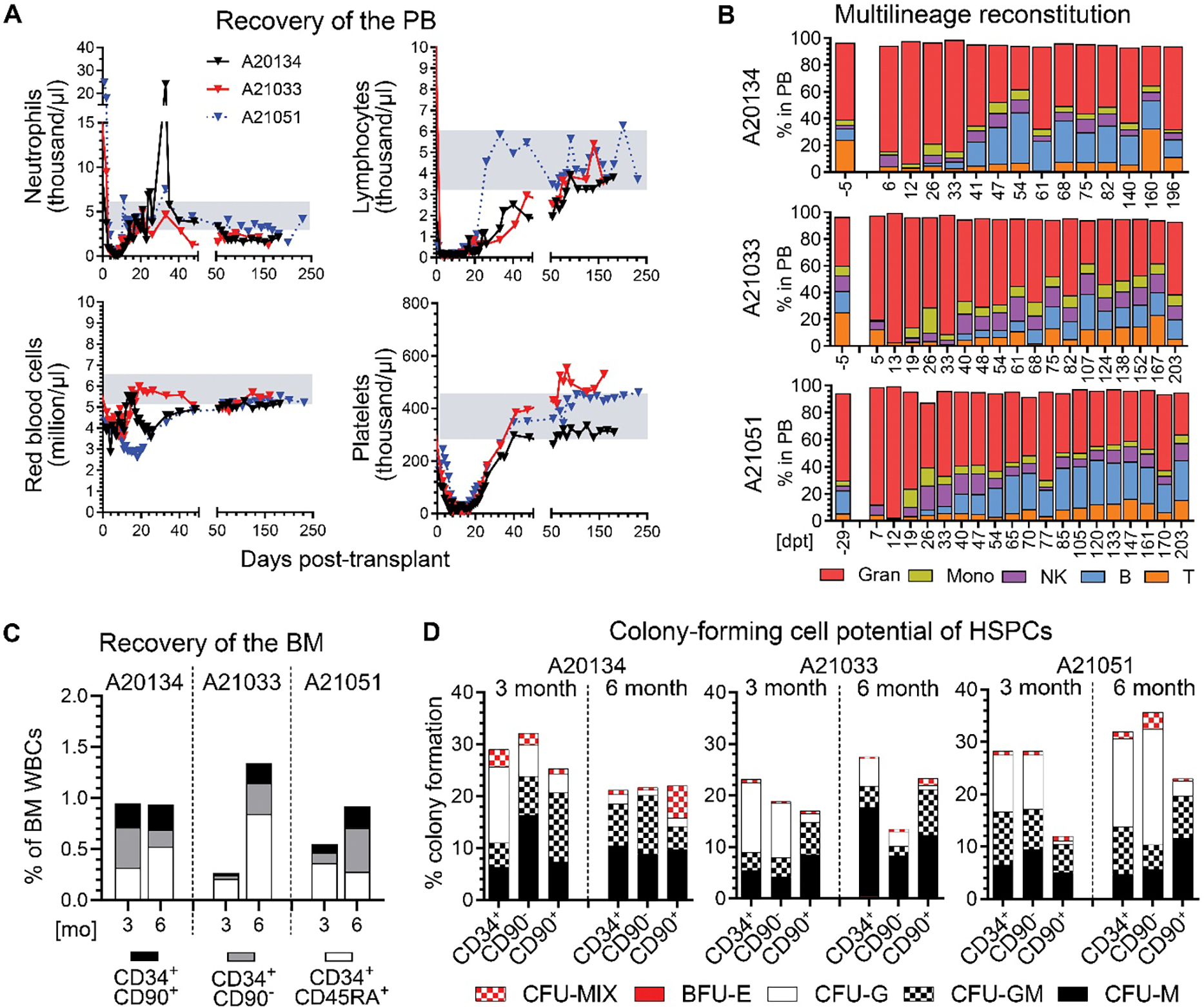
Recovery of the peripheral blood and bone marrow compartments after transplantation of base-edited HSCs. (**A**) Neutrophil and lymphocyte values (top row) as well as red blood cell and platelet values (bottom row) for the NHPs from day 0 to 40 and day 50 to 250 post-transplant. The gray shaded area on each graph indicates the range of normal values in naïve control animals. (**B**) Composition of PB lineages for each animal, assessed before and serially post-transplant. Abbreviations: Gran = Granulocytes; Mono = Monocytes; NK = NK cells; B = B cells; T = T cells. (**C**) Quantification of HSPC subsets in the BM at 3- and 6-months (mo) after transplant. (**D**) Percentage colony formation of engrafted and FACS-purified HSPC subsets from each animal at 3- and 6- months post-transplant. Abbreviations: CFU = colony-forming unit; G = granulocyte; M = monocyte/macrophage; BFU = burst forming unit; E = erythrocyte; MIX = myeloid + erythroid.

To confirm multilineage long-term engraftment potential of base-edited CD34^+^CD90^+^ HSCs, transplanted animals were closely followed for up to 200 days, monitoring their complete blood cell counts in the peripheral blood (PB) as well as taking bone marrow (BM) aspirates at 3- and 6-months post-transplant (Fig. 3). Neutrophil and platelets in the PB recovered within 8–9 and 19–22 days post-transplant (>500 neutrophils per μl, > 50K platelets per μl), respectively (table S1). Granulocytes, lymphocytes, red blood cells, as well as platelets reached the range of normal (21) within 30–60 days post-transplant and remained stable thereafter (Fig. 3A). Longitudinal flow-cytometric assessment of PB lineages showed the expected initial granulocytes bias during early recovery within the first month post-transplant (Fig. 3B). Lymphocytes recovered shortly after and the blood fully reestablished pre-transplant composition after 5–6 months.

Next, we analyzed the recovery of the BM stem cell compartment (Fig. 3C) and compared the composition to previously reported naïve animals (19). Flow-cytometric analysis showed full reconstitution of BM CD34^+^ HSPCs including all phenotypic CD34^+^ subsets within 6 months post-transplant (Fig. 3C). HSPCs in A20134 recovered at 3 months post-transplant because this animal received more CD34^+^CD90^+^ HSCs per kg body weight than A21051 or A21033 (table S1). Three- and 6-month post-transplant engrafted CD34^+^ HSPCs and subsets were tested using CFC assays to assess erythro-myeloid differentiation potentials (Fig. 3D). Overall colony-forming potential (height of bar) as well as composition of colonies (pattern) was within the range of normal, confirming the ability of CD34^+^ HSPCs and CD34^+^ subsets to realize erythroid, myeloid, as well as erythro-myeloid cell fates.

### Stable and persistent contribution of base-edited CD34^+^CD90^+^ HSCs to all lineages in the PB and BM

**Fig. 4.**
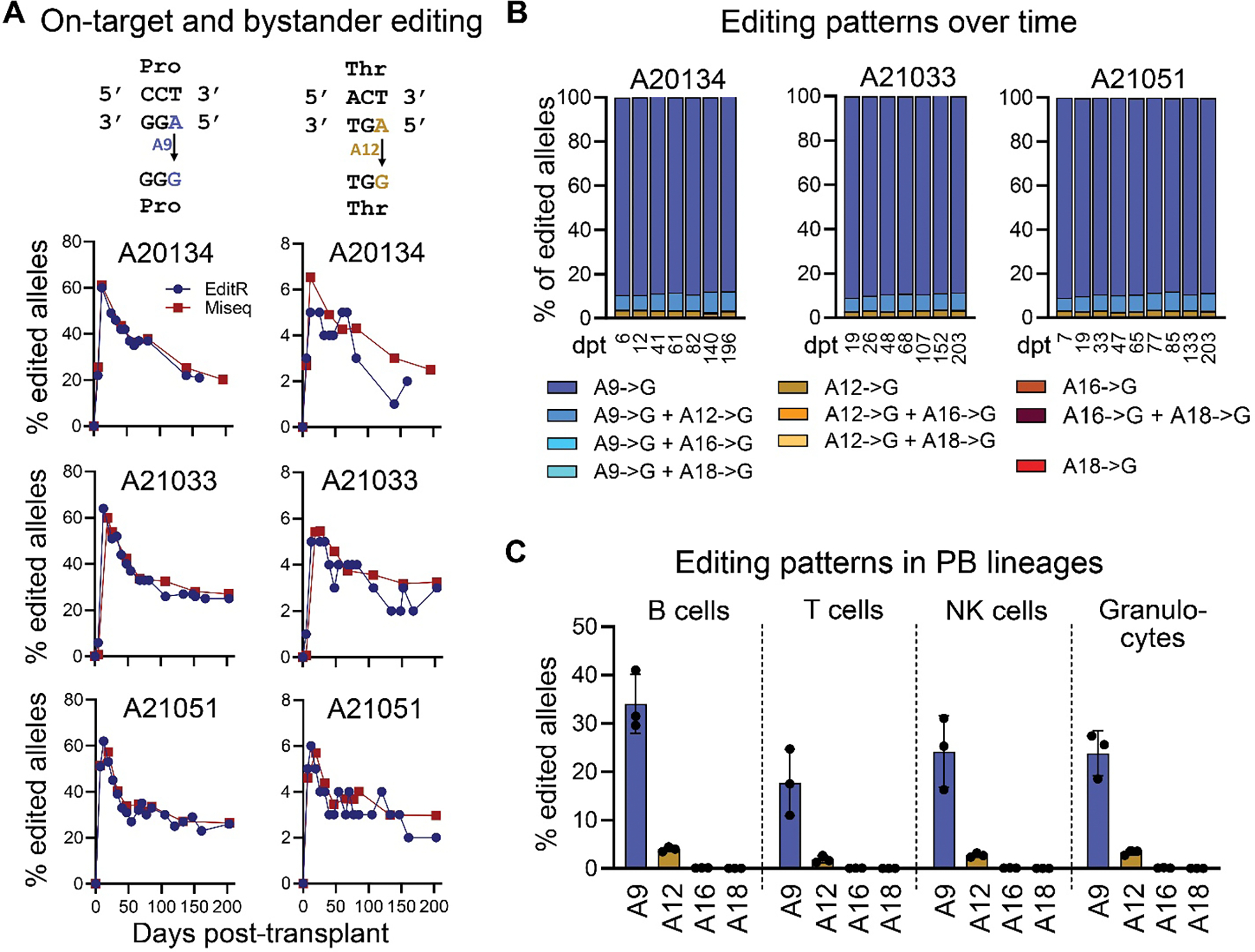
Longitudinal tracking of on-target and bystander editing in the peripheral blood. (**A**) Longitudinal tracking of the base editing frequency at adenines A9 (left) and A12 (right) in PB WBCs of each animal using Sanger sequencing (EditR, blue) and NGS (red). (**B**) Combination of editing events in PB WBCs over time using NGS. (**C**) Frequency of base editing in FACS-purified PB lineages 6 months post-transplant as determined by NGS at all four adenines in the protospacer.

To determine the contribution of base-edited HSCs to hematopoietic recovery as well as steady-state hematopoiesis, longitudinal PB samples were taken and analyzed using Sanger sequencing and NGS. Assessment of A9 editing in bulk white blood cells (WBCs) from the PB showed a spike in editing 10–20 days after transplant and a gradual decline in the first 3 months with long-term persistence of editing at 20–30% until the end of study (Fig. 4A). Sanger sequencing as well as NGS data closely matched throughout the entire follow-up. Bystander editing at position A12 similarly spiked early, declined, and plateaued at 2–4% long-term. Because detection of A12 editing was highly variable using Sanger sequencing, whereas NGS showed less variability across time-points, we analyzed bystander editing at A12, A16 and A18 only using NGS. Editing at A12 was primarily found in conjunction with editing at A9 and was rarely found to occur independently (Fig. 4B). Lastly, bystander editing at positions A16 and A18 was too low to be reliably detected even with NGS and within the range of background noise seen for unedited control samples taken from the same animals before transplant (fig. S1).

Next, we analyzed FACS-purified PB lineages 6 months post-transplant to confirm multilineage contribution of base-edited CD34^+^CD90^+^ HSCs (fig. S2A, Fig. 4C). A9 editing was detected across myeloid and lymphoid lineages (Fig. 4C). Detailed assessment of all four adenines with NGS further showed low or undetectable frequencies of bystander editing across all lineages.

**Fig. 5.**
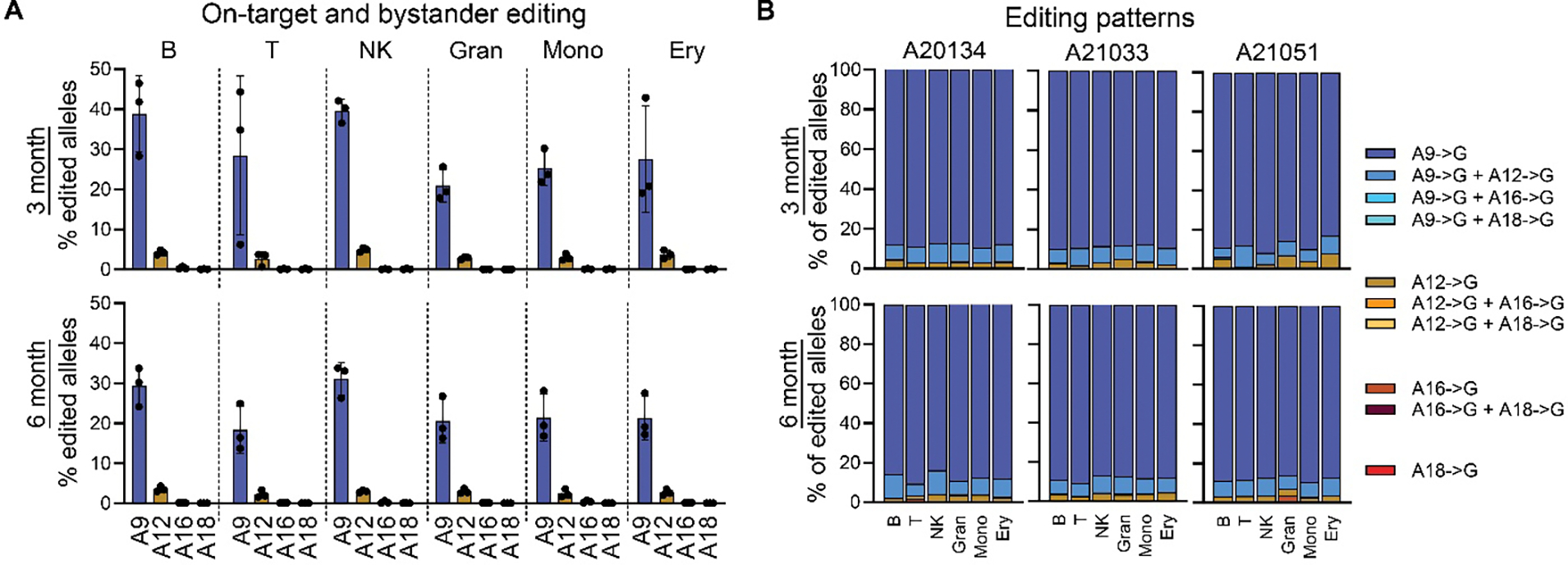
Editing in bone marrow lineages. (**A**) Frequency of base editing in FACS-purified BM lineages 3- and 6-months post-transplant were determined at all four adenines in the protospacer using NGS. Abbreviations: Gran = Granulocytes; Mono = Monocytes; NK = NK cells; B = B cells; T = T cells; Ery = CD71^+^ cells. (**B**) Combinations of editing events in FACS-purified BM lineages 3- and 6-months post-transplant, determined by NGS.

To further assess the contribution of base-edited CD34^+^CD90^+^ HSCs to BM-resident erythroid precursors (CD71^+^ progenitors), BM samples were collected at 3- and 6-months post-transplant, phenotypic lineages were FACS-purified (fig. S2B), and editing analyzed using NGS (Fig. 5A). Base editing in BM lineages ranged from 20–40% at the earlier 3-month timepoint and stabilized at 25–35% across lineages after 6 months (Fig. 5A). CD71^+^ erythroid progenitors on average showed similar editing efficiency as in all other lineages, suggesting no impairment of *HBB* base editing on differentiation. As seen in PB lineages, editing was predominantly found at position A9, and low-frequency bystander editing at A12 occurred most commonly alongside A9 editing (Fig. 5B). These data confirm that base editing of NHP CD34^+^CD90^+^ HSCs at *HBB* did not affect long-term multilineage differentiation, and base-edited HSCs contributed evenly to all lineages in the PB and BM throughout the follow-up period.

### Long-term engraftment of base-edited CD34^+^CD90^+^ HSCs in the bone marrow stem cell compartment

**Fig. 6.**
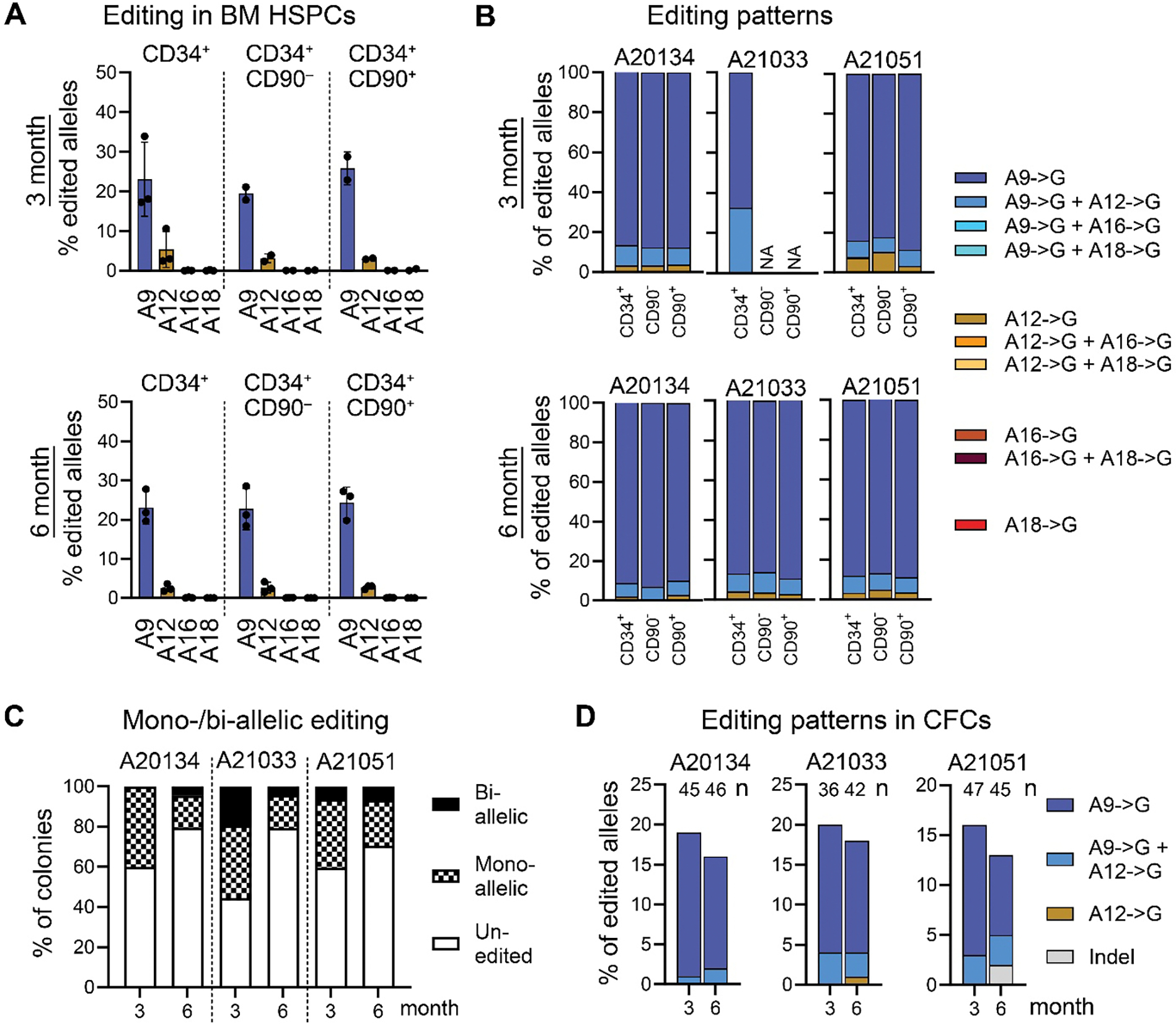
Engraftment and persistence of base-edited HSPCs in the bone marrow. (**A**) Frequency of base editing in FACS-purified BM HSPC subsets at all four adenines in the protospacer in samples collected at 3 months (left) and six months (right) post-transplant, determined by NGS. (**B**) Combinations of editing events in FACS-purified BM HSPC subsets 3- and 6-months post-transplant, as determined by NGS. (**C and D**) Assessment of mono- and biallelic editing **(C),** and editing patterns of single colonies from Fig. 3D (**D**), all assessed by Sanger sequencing (EditR) at position A9.

To provide a life-long cure, base-edited CD34^+^CD90^+^ HSCs need to home to and stably engraft in the BM stem cell compartment. To confirm successful engraftment and long-term persistence of base-edited CD34^+^CD90^+^ HSCs, BM samples were taken 3- and 6-months post-transplant, CD34^+^ HSPCs were MACS-purified, and phenotypic CD34^+^ subsets were isolated using FACS (fig. S1B). Genomic assessment of FACS-purified HSPC subsets demonstrated similar frequencies of editing across all populations as previously seen in PB and BM lineages (Fig. 6A). No differences were found in the frequency and pattern of bystander editing in any HSPC subsets in comparison to previously analyzed lineages in the PB and BM (Fig. 6B).

Next, we quantified the frequency of mono- and biallelic editing on a clonal level by harvesting individual CD34^+^-derived colonies (Fig. 3D) using Sanger sequencing (Fig. 6, C and D). Base editing at position A9 was found in all three animals in more than 40% of colonies after 3-months and 20% after 6 months, matching the frequency of editing observed in bulk CD34^+^ HSPCs, HSPCsubsets as well as lineages (Fig. 6C). About 80% of edited colonies demonstrated mono-allelic editing in all three animals at the 6-month time point, resembling the pattern of editing initially seen in the infusion product (Fig. 2B). Lastly, editing primarily occurred at position A9 with and without the bystander editing at position A12 (Fig. 6D). Of note, in a total of 261 colonies only two indels were found, both from A21051, at the 6-month timepoint. In summary, ex vivo base-edited CD34^+^CD90^+^ HSCs successfully homed, engrafted, reconstitution the CD34^+^ HSPC pool, and contributed long-term to the BM as well as PB without any change in their phenotypic appearance or functional potential.

### Rare base editing events at the limit of detection

To assess bystander editing of our ex vivo base editing approach, NGS data collected throughout the entire follow-up of the animals were comprehensively analyzed for previously reported rare events. Adenine base editors have previously been shown to have activity at nearby cytosines leading to deamination (22). Therefore, we specifically analyzed the activity of ABE8e at the four cytosines C3, C5, C6, and C8 (fig. S3). Putative substitution at all four cytosines were determined by analyzing all collected NGSs datasets from PB, BM, and HSPCs. Low frequencies of substitutions at cytosines above background noise (indicated for each position and base individually in unedited control samples from the same animals before transplant) were found in positions C5, C6, and C8 but not C3. Substitutions were predominantly C>T and C>G in all three affected positions and rarely C>A. The frequency of substitution remained below 0.1% across all time points and was not affected by the source or type of sample (bulk, lineage purified) analyzed.

**Fig. 7.**
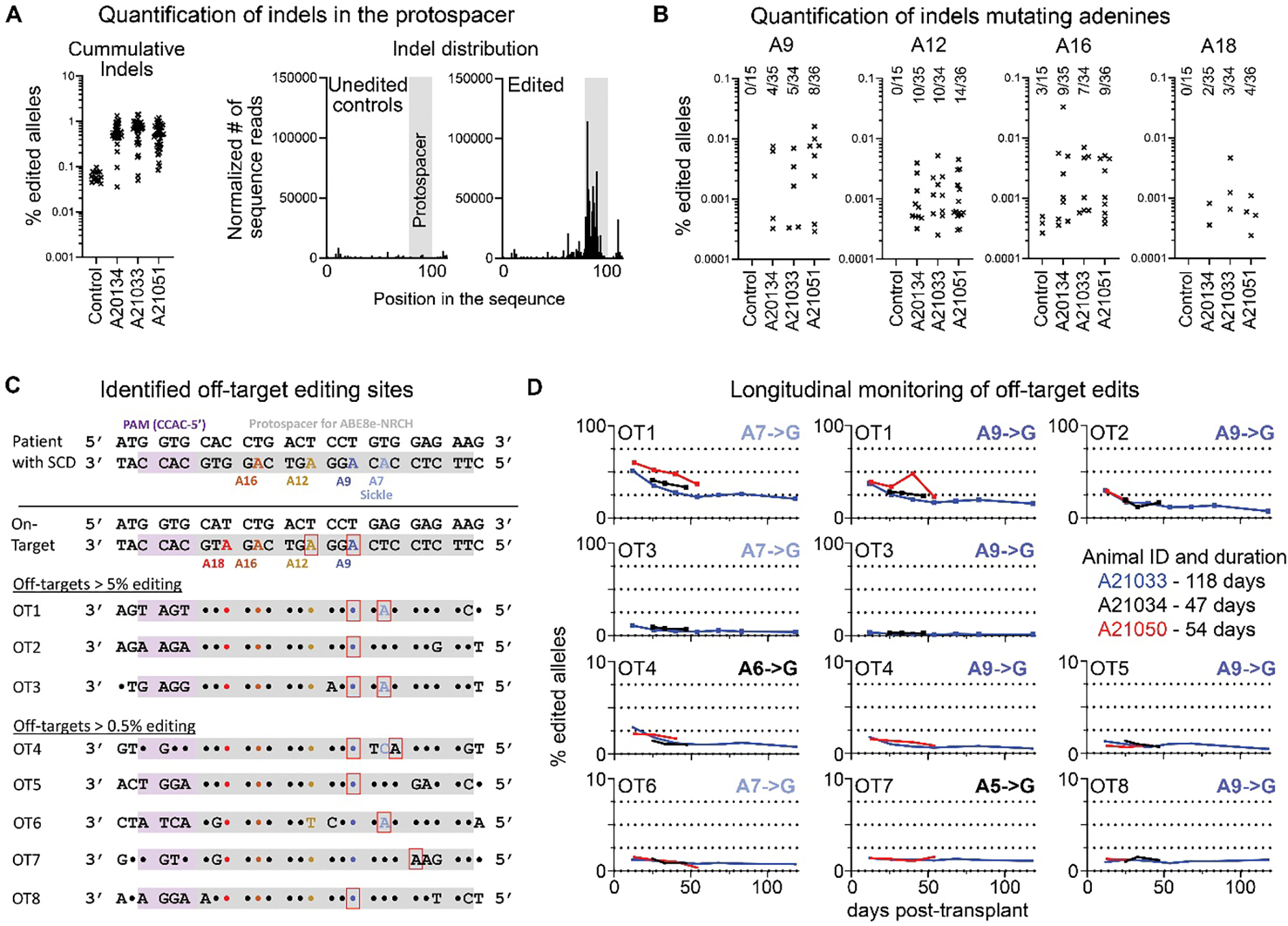
Analysis of rare base editing events and off-target editing. (**A**) Cumulative frequency of indels detected in the protospacer region by NGS in PB WBC samples taken before editing (controls) and after transplant in the three animals (left). Localization of indels quantified in the first graph relative to the protospacer (grey vertical bar). Middle graph shows indels detected in samples taken before editing to establish background noise introduced by PCR and sequencing in PB WBC samples taken before editing. Right graph shows a summary of all indels detected in PB WBC samples taken from all three animals post-transplant. (**B**) Indel frequency at A9, A12, A16, and A18, as assessed by NGS. The numbers above each column indicate the number of samples with indels present and total number of samples analyzed. (**C**) Off-target sites showing A-to-G editing were identified using rhAMPseq. Editing sites are highlighted with red boxes. (**D**) Frequency of A-to-G editing at the off-target sites in PB WBCs over time in the three animals as summarized in (C).

Next, we looked at the potential formation of indels across the entire protospacer in bulk as well as on the single cell level (Fig. 7A, fig. S4). No indels were found to occur with frequencies greater than 0.15%, and the top 4 most prevalent indels showed a duplication of 7–8 bps (base pairs) within the protospacer (fig. S4A). The cumulative frequency of all detected indels was below 1% in all three animals at all times, and indels were exclusively found within the protospacer area throughout the entire follow-up (Fig. 7A, fig. S4B). Lastly, a more specific assessment of events at the adenines confirmed that indel formations were irregularly found at frequencies below 0.01% in all three animals (Fig. 7B).

Lastly, we assessed off-target editing. The top thousand putative off-target sites were bioinformatically determined using Cas-OFFinder, 951 of which were amenable to pooled rhAMPseq, and a total of 16 longitudinally collected PB WBC samples from all three animals were amplified by pooled rhAMPseq primers, followed by Illumina sequencing. Analysis showed significant (p<0.05) A-to-G off-target editing in eight sites (Fig. 7C, table S2). Longitudinal assessment of all eight sites showed a slight decline in the frequency but persistence of edited alleles over time, similar to the persistence of cells harboring the target edit, suggesting there is no benefit or disadvantage of edits for blood cells (Fig. 7D, table S2). Detailed analysis of the off-target sites determining the nearest annotated or predicted gene using HOMER (Hypergeometric Optimization of Motif EnRichment) (23) showed no association with known oncogenes and localization of edits primarily in intronic or non-coding regions (table S3). In summary, a variety of very rare low-frequency off-target editing and indel events were found to occur in the protospacer and persisted long-term, without evidence for positive selection.

## DISCUSSION

Here we demonstrated efficient and persistent base editing of NHP CD34^+^CD90^+^ HSCs for SCD treatment. Base-edited CD34^+^CD90^+^ HSCs showed no impairment in homing, long-term engraftment, or multilineage differentiation in the NHPs. The editing efficiency of the adenine at position A9 in NHP HSCs was almost identical to the previously observed editing in human HSCs from patients with SCD to generate the Makassar variant, in which the target nucleotide encoding the SCD variant is in position A7, and A9 is a silent bystander edit. These data confirm that the Makassar base editing strategy is similarly efficient in NHP HSCs, allowing for the pre-clinical modeling of this treatment strategy with translational potential for patients with SCD.

Genome editing tools have rapidly evolved in the last decades. Although most of the technologies have been tested in vitro as well as in vivo in rodent models (24–28), the clinical translation of newer editors is slowed by the complexity and limited availability of appropriate animal models. In particular, assessing editing strategies that involve autologous transplantation of HSCs is laborious and requires complex infrastructure even in the pre-clinical setting. Studies in NHPs help to assess the long-term efficiency of adenine base editors, evaluate feasibility, and demonstrate scalability of procedures for the translation into clinical applications. The use of ABE8e for gene editing of HSCs ex vivo demonstrated high efficiency with reduced variability in comparison to previous studies using CRISPR (Clustered Regularly Interspaced Short Palindromic Repeats)/Cas9 (29) or TALENs (Transcription activator like effector nucleases) (30). We also observed a high degree of reproducibility in vivo across the three animals with long-term persistence of gene-edited cells above the reported threshold of 20% that is needed for substantial phenotypic benefit. No impact of *HBB* editing was noted during the ex vivo processing and editing of HSCs, quality control of infusion products for the phenotype and function of HSC subsets, or neutrophil/platelet/RBC recovery and overall health of the animals, indicating that base editing of *HBB* is scalable from rodent xenograft models to NHPs, feasible, and well tolerated by HSCs.

We chose to edit the β-globin gene in NHPs and mimic the introduction of the therapeutically-relevant Makassar mutation for treatment of SCD in patients. However, because an NHP model of SCD is not available, we instead monitored silent bystander editing at an adenine two base pairs downstream of the pathogenic SCD mutation as a surrogate for the editing efficiency at the *HBB* sickle locus (12). The protospacer placement and length match the previously described editing strategy in human patient samples, which allow for the side-by-side comparison of the editing efficiency in sickle patient HSCs. Despite the needed change in the guide RNA to accommodate the two-base pair difference in the protospacer between patients with SCD and NHPs (the sickle cell disease-causing variant itself and one additional silent polymorphism), almost identical editing efficiency was observed in the homologous adenines of human and NHP HSCs, providing evidence that this editing strategy closely resembles what could be achieved in the clinic. We propose that monitoring surrogate bystander edits in NHPs could be broadly useful as genome editing medicines are optimized for clinical use. Lastly, the ease of making adjustments to the gRNA and preserving activity across species further highlights the versatility of this technology and the potential use for other hematological diseases and disorders where ABEs may be applicable.

The editor and guide RNAs in our study were delivered using electroporation, a widely used method for mRNA and protein delivery and the current gold standard for ex vivo HSC gene therapy in clinical trials. Furthermore, we used TBI for conditioning, nowadays a less frequently used conditioning strategy for autologous HSC gene therapy treatments currently favoring busulfan-mediated ablation of endogenous bone marrow HSCs (31–33). Methods to deliver genome editors to HSCs in vivo are urgently needed to avoid the genotoxic bone marrow ablation with either TBI or busulfan and complex transplantation procedures that must otherwise be used. Many strategies to achieve this are still being explored and include the use of non-integrating viral methods such as adenoviruses and adeno-associated viruses (AAVs) (27, 34). Strategies to transiently deliver genome editor RNAs or proteins such as virus-like particles, lipid nanoparticles, and polymer nanoparticles are particularly attractive because they can more easily avoid unnecessary prolonged expression of genome editors (35, 36). A wide range of these technologies has already been used in vivo for non-hematological applications (25, 37–39) and more recently also shown promise in editing blood cells (34).

Off-target editing was previously observed at more than 50 sites using this base-editing strategy to introduce the Makassar variant in patient-derived human HSCs (12). Here, we identified a total of 8 sites in NHP PB WBCs demonstrating stable off-target editing without any evidence of selective outgrowth or survival benefits/disadvantages. Although none of these identified sites posed an obvious danger to cell health, it is possible that the higher frequencies of off-target editing conferred by the particularly active ABE8e deaminase could lead to toxicity or disruption of the ability of transplanted HSCs to reconstitute a normal hematopoietic system. We assessed whether the high editing activity from ABE8e-NRCH would lead to toxicity or issues in the reconstitution of hematopoietic lineages and did not observe any such issues. Nevertheless, before transitioning this into a clinical product, additional studies should test modulation of the editor domains to minimize off-target editing propensity while maintaining sufficiently high on-target editing efficiency. Such studies should ideally be conducted in human cells to ensure that the off-target risks will most closely represent those that will be present in the clinic.

A recent study highlighted the byproducts that can be generated following base editing in human HSCs (40), including deletions, translocations, and transcriptional changes. Notably, all base editors yielded far fewer undesired byproducts relative to Cas9 nuclease, and the adenine base editor showed a more favorable profile relative to the cytidine base editor, with no translocations detected after adenine base editing. The use of Cas9 nuclease targeting the *BCL11A* erythroid-specific enhancer in cells transplanted in patients has already shown positive outcomes (41). We expect that the adenine base-editing strategy we assess here will have an even more favorable safety profile due to the reduction of undesired and potentially genotoxic byproducts.

Although we here demonstrate that adenine base editing of *HBB* in HSCs and autologous transplantation of gene-edited cells is efficient in NHPs at this particular locus, additional studies will be needed to confirm overall safety for the clinical translation. Limitations that will need to be addressed include the assessment of clonality and potential outgrowth of dominant clones over time, toxicities associated with the base editors including immune responses, or genomic alterations such as chromosomal rearrangements and large deletions as byproducts of the editing process as seen for many Cas9-mediated strategies. Furthermore, off-target editing sites discovered in these NHPs are not directly transferrable to human patients with SCD due to the minor modification needed in the gRNA to match the NHP genome and account for the lack of the SCD-causing mutation leading to putative off-targets that were not described in the human genome, therefore limiting the application of off-target results generated in this study for a clinical translation of drawing broadly applicable conclusions for all genomic targets. Comprehensive on- and off-target studies will be needed for every locus targeted with ABEs to carefully assess the safety.

In summary, our proof-of-concept study using ABEs for ex vivo HSC gene therapy in a pre-clinical NHP model demonstrated scalability, feasibility, reproducibility, as well as high long-term efficiency, many pre-requisites for the clinical translation of base editing. Rapid short-term recovery, durable engraftment of *HBB*-edited CD34^+^CD90^+^ HSCs, and no occurrence of adverse events in all three animals, suggest that base editing is well tolerated by HSCs, has little effect on the homing ability, does not impact the cells short- or long-term engraftment, and does not to a change in multilineage output.

## MATERIALS AND METHODS

### Study design

This study was designed to assess the engraftment of autologous base edited hematopoietic stem cells and repopulation of the hematopoietic system in a nonhuman primate model, which could serve as a treatment for sickle cell disease. CD34^+^CD90^+^ cells enriched for hematopoietic stem cells were electroporated with base editor mRNA and guide RNA before autologous transplantation into TBI-conditioned NHPs, which were monitored over a 6-month period for multilineage engraftment and on- and off-target editing. All experimental procedures performed were reviewed and approved by the Institutional Animal Care and Use Committee of the Fred Hutchinson Cancer Center (Fred Hutch) and UW (Protocol No. 3235–01). Sample sizes were not predetermined by a statistical method. Investigators were not blinded to sample identity because all three animals in the study received the same treatment, with control samples collected before experimental procedures began for each animal. Inclusion criteria specified that only healthy juvenile rhesus macaques were used in this study, with no additional exclusion criteria.

### Animals

Healthy juvenile rhesus macaques (Table S1) were housed at the University of Washington (UW) National Primate Research Center (WaNPRC) under conditions approved by the American Association for the Accreditation of Laboratory Animal Care. This study was carried out in strict accordance with the recommendations in the *Guide for the Care and Use of Laboratory Animals of the National Institutes of Health* (“The Guide”), and monkeys were randomly assigned to the study. This study included at least twice-daily observation by animal technicians for basic husbandry parameters (for example, food intake, activity, stool consistency, and overall appearance), as well as daily observation by a veterinary technician and/or veterinarian. Animals were housed in cages approved by “The Guide” and in accordance with Animal Welfare Act regulations. Animals were fed twice daily and were fasted for up to 14 hours before sedation. Environmental enrichment included grouping in compound, large activity, or run-through connected cages, perches, toys, food treats, and foraging activities. If a clinical abnormality was noted by WaNPRC personnel, standard WaNPRC procedures were followed to notify the veterinary staff for evaluation and determination for treatment as a clinical case. Before all procedures, animals were sedated by administration of ketamine HCl and/or telazol and supportive agents for balanced anesthesia (such as diazepam and midazolam). After sedation, animals were monitored according to WaNPRC standard protocols. WaNPRC surgical support staff are trained and experienced in the administration of anesthetics and have monitoring equipment available to assist with electronic monitoring of heart rate, respiration, and blood oxygenation; audible alarms and digital readouts; monitoring of blood pressure, temperature, etc. For minor procedures, the presence or absence of deep pain was tested by the toe-pinch reflex, and the absence of response (leg flexion) to this test indicated adequate anesthesia. In cases of general anesthesia, similar monitoring parameters were used, and anesthesia was tested by the loss of palpebral reflexes (eye blink). Analgesics (generally buprenorphine with meloxicam or buprenorphine slow release) were provided as prescribed by the clinical veterinary staff for at least 48 hours after the procedures and could be extended at the discretion of the clinical veterinarian based on clinical signs. Autologous NHP HSC transplantation, G-CSF/SCF treatment for bone marrow priming, collection of G-CSF/SCF primed whole BM, CD34 enrichment, and base editing of *HBB* with ABE8e-NRCH mRNA and synthetic gRNA were conducted consistent with our previously published protocols (19, 20, 42). In parallel to cell processing, macaques were conditioned with myeloablative TBI of 1020 cGy from a 6-MV x-ray beam of a single-source linear accelerator located at the Fred Hutch South Lake Union Facility (Seattle, WA); irradiation was administered as a fractionated dose over the 2 days before cell infusion. During irradiation, animals were housed in a specially modified cage that provided unrestricted access for the irradiation while simultaneously minimizing excess movement. The dose was administered at a rate of 7 cGy/min delivered as a midline tissue dose. Granulocyte colony-stimulating factor was administered daily from the day of cell infusion until the animals began to show onset of neutrophil recovery. Supportive care, including antibiotics, electrolytes, fluids, and transfusions, was given as necessary, and blood counts were analyzed daily to monitor hematopoietic recovery.

### CD34 enrichment, CD90 HSC FACS, and in vitro cultures

Primed NHP BM was harvested, enriched, and cultured as previously described (42, 43). Briefly, before enrichment of CD34^+^ cells, red blood cells were lysed in ammonium chloride lysis buffer, and white blood cells were incubated for 30 min with the 12.8 immuno-globulin M anti-CD34 antibody and then washed and incubated for another 30 min with MACS anti–immunoglobulin M microbeads (Miltenyi Biotec). The cell suspension was run through magnetic columns to enrich for CD34^+^ cell fractions with a purity of 60 to 80%, which was confirmed by flow cytometry (see Flow Cytometry and FACS section). CD34^+^CD90^+^ and CD34^+^CD90^–^ cell fractions were purified using a FACS Aria II (BD Biosciences).

### CFC assay

For CFC assays, 1000 to 1200 sorted cells were seeded into 3.5-mL ColonyGEL 1402 (ReachBio). Hematopoietic colonies were scored after 12 to 14 days. Arising colonies were identified as colony-forming unit (CFU) granulocyte (CFU-G), CFU macrophage (CFU-M), CFU granulocyte-macrophage (CFU-GM), and burst-forming unit-erythrocyte (BFU-E). Colonies consisting of erythroid and myeloid cells were scored as CFU-MIX.

### Colony PCR

Individual colonies were picked and stored in QuickExtract DNA Extraction Solution (Lucigen, QE09050). Colonies were heat lysed at 65°C for 20 minutes, 99°C for 10 minutes, and then cooled down to be stored at −20°C. 5 μL of the lysed colony was used as a template for PCR amplification using the forward and reverse primers listed in Table S4. Resulting amplicons were run on a 1.2% agarose gel with 1 kb ladder.

### Base editor in vitro mRNA transcription

Similar to previous in vitro transcription of prime editor mRNAs (44), plasmids were cloned to encode an inactivated T7 promoter followed by a 5′ untranslated region (UTR), Kozak sequence, the coding sequence of ABE8e-NRCH, and a 3′ UTR. T7 promoter inactivation prevents potential transcription from circular plasmid template during mRNA generation. These components together were PCR-amplified with NEBNext polymerase (New England BioLabs) using primers that correct T7 promoter inactivation and append a 120-nt poly(A) tail to the 3′ UTR. The resulting PCR product was purified with the QIAquick PCR Purification Kit (QIAgen) and served as a template for subsequent in vitro transcription. ABE8e-NRCH mRNA was transcribed from these templates using the HiScribe T7 High-Yield RNA Synthesis Kit (New England BioLabs) with co-transcriptional capping by CleanCap AG (TriLink Biotechnologies) and full replacement of UTP with N1-Methylpseudouridine-5′-triphosphate (TriLink Biotechnologies). Transcribed mRNA was precipitated in 2.5 M final concentration of lithium chloride (7.5M stock from Thermo Fisher Scientific), washed twice in 70% ethanol, then dissolved in nuclease-free water. The resulting mRNA was quantified with a NanoDrop One UV-Vis spectrophotometer (Thermo Fisher Scientific) and was aliquoted and stored at −80°C.

### Base editing

ABE8e-NRCH mRNA was produced by the Liu Lab and chemically modified gRNA (2′-O-methyl analogs and 3′ phosphorothioate internucleotide linkages at the first three 5′ and 3′ terminal RNA residues) was custom-ordered from Synthego and is listed in Table S4. Briefly, FACS-purified CD34^+^CD90^+^ cells were electroporated using the BTX ECM830 electroporator (Harvard Apparatus) at 3×10^6^ living cells per 2 mm cuvette for 5 ms at 250V. For the electroporation, cells were resuspended in 100 μl BTX buffer supplemented with 12 μg ABE8e-NRCH mRNA and 12 μg Makassar sgRNA per cuvette.

### Flow cytometry and FACS

Antibodies used for analysis and FACS sorting of NHP cells are listed in Table S5. Dead cells and debris were excluded by forward scatter (FSC)/ side scatter (SSC) gating. Flow cytometric analysis was performed on a Symphony A5 Iiu and FACS was performed on aFACSAria Iiu (BD Biosciences). Cells for in vitro assays as well as autologous NHP stem cell transplants were FACS-purified right after CD34^+^ enrichment using a FACS Aria IIu cell sorter (BD Biosciences) and sort purity assessed by recovery of sorted cells.

### Sanger sequencing and EditR analysis

PCR amplicons were sequenced by Sanger sequencing and analyzed using the web-based EditR program (Table S1) (45). Indels identified via Sanger sequencing were flagged during EditR analysis when the sequence degraded within the protospacer. Samples suspected of containing indels were then Sanger-sequenced using primers from both directions and analyzed using TIDE (default settings except for max indel size set to15bp) (46).

### NGS and data analysis

For NGS, genomic DNA was extracted using QIAamp DNA micro kit (Qiagen) and processed by PCR amplification using the primers described in Table S4. Libraries were prepared using Illumina barcoded, 2×150 base-pair (bp), pair-end and run on the Miseq platform (Illumina). The paired-end reads were merged using Paired-End read Merger (PEAR) with default parameters (47). A custom Python script was used for the bioinformatics analysis of the sequencing data. The reads were filtered if they had more than 2 bases with low quality scores. The merged reads were aligned to the start primer and the end primer sequences allowing for 2 mismatches with no insertions and deletions (indels). The reads with good primer alignment were then aligned to the wild-type locus sequence using Needle, a Needleman-Wunsch aligner from the EMBOSS Suite (http://emboss.sourceforge.net/apps/release/6.6/emboss/apps/needle.html) (48). The reads were grouped based on the alignment pattern into the following: (i) reads that match the wild-type sequence (ii) reads with the Makassar base edits (iii) reads with the off-target base edits and (iv) reads with different substitution and indel patterns and the frequencies of the groups were calculated.

### rhAMPseq probe design

The CasOFFinder webtool (http://www.rgenome.net/cas-offinder/) (49)was used to nominate candidate off-target sites in the Rhesus macaque genome. We extracted genomic loci for all sites in the Macaca mulatta genome with four or fewer mismatches to the target sequence “TTCTCCTCAGGAGTCAGATG” and no bulges. Due to the relative promiscuity of the NRCH Cas9 PAM (protospacer adjacent motif) sequence (18), which can recognize PAMs outside of the NRCH consensus, we used “NNN” as the permitted PAM sequence so as not to limit the search. This list included 2 sites with one mismatch to the spacer region, 23 sites with two mismatches, 328 sites with three mismatches, and 3971 sites with 4 mismatches. We generated a list of 1000 sites for rhAMPSeq analysis, including all sites with one to three mismatches and the top 647 sites with four mismatches as ranked by the mitOfftargetScore output by CasOFFinder. We converted the nucleotide coordinates from the rheMac3 genome assembly available in CasOFFinder to the Mmul_10 genome assembly that can be used for rhAMPSeq design with IDT using the UCSC Genome Data Browser LiftOver function (https://genome-asia.ucsc.edu/cgi-bin/hgLiftOver) (50). IDT automated design of rhAMPSeq primers was used to generate the final pool, which was able to multiplex 951 off-target sites.

### rhAMPseq and sequencing

A total of 16 specimens were used for rhAMPseq. DNA from PB WBCs were extracted using DNA extraction kits (Qiagen). Extracted DNA was processed to generate targeted rhAMPseq PCR amplicons using the custom rhAMPseq probe library designed above following the manufacturer’s instructions (IDT). Index rhAMPseq libraries were generated using rhAMPSeq Index primers (IDT) following the manufacturer’s instructions. Indexed rhAMPseq libraries were sequenced in the British Columbia Cancer Research Center (Vancouver, Canada) using the Illumina NovaSeq X platform generating paired-end reads of 150bp length.

### rhAMPseq data analysis

The raw reads were merged using Paired-End read Merger (PEAR) with default parameters (47). A custom Python script was used for the bioinformatics analysis of the sequencing data (51). The reads with low quality base scores were filtered before aligning them. The merged reads for each sample were aligned to the forward and reverse primers and all the off-target sequences from the Cas-OFFinder output. Based on the alignment, the frequency of all the nucleotides (A, C, G, T) at a position and the base error rate, was calculated for all the positions in the amplicon sequence for each off-target sequence in every sample.

For each sample, we only analysed off-target sites with more than 1000 reads. The editing window in the candidate protospacer alignments within the amplicons for each of the off-target sites were assessed for editing using custom R scripts (51). For the animals with pre-treatment samples, mutations that were present in the pre-treatment time point at frequencies above 10% and also present in all the other timepoints were classified as single nucleotide polymorphisms (SNPs) and excluded. We calculated a mean baseline background error rate and standard deviation for each of the pre-treatment samples using the base error rates for each of the positions in the protospacer sequences for all the respective off-targets in the sample. If the protospacer sequence had a SNP, that particular base in the protospacer was excluded for the background error rate calculations.

We set a threshold of 2 x standard deviation to identify mutations of interest in the off-target sequences from all the samples excluding the pre-treatment. Every position in the putative off-target protospacer binding site which had a mutation and met the threshold criteria (>0.5%) was considered a potential candidate.

We made a table of the mutation frequencies of all the potential candidate positions from all timepoints for each of the 3 animals in the study and employed a one-sided t-test to calculate p values for each of the candidate positions. We identified 8 off-target positions that were statistically significant (p<0.05) in comparison to the pre-treatment samples.

For genomic feature analysis of the off-target positions, we used the HOMER suite of tools (23). We used the annotatePeaks.pl program to annotate the off-target positions based on their chromosomal co-ordinates in the Rhesus genome (rheMac10) (23).

### Statistical analysis

Statistics were only applied to the rhAMPseq data as described above. No other statistical tests were applied to the presented data.

## Supplementary Material

Supplemental Materials, Tables, and Figures

Data Files S1

MDAR Reproducibility Checklist

## Data Availability

All data associated with this study are present in the paper or supplementary materials. NGS and rhAMPseq data can be found under BioProject ID PRJNA1036686 on the NCBI sequence read archive (SRA). The code used in this study is available at https://github.com/KiemLab-RIS/Makassar_project and has been deposited in Zenodo at: 10.5281/zenodo.15190299 (51).
