# Supplementary material for: Engraftment and persistence of HBB-base-edited hematopoietic stem cells in nonhuman primates
Source: Sci Transl Med. Author manuscript; Available in PMC 2025 Oct 3. (PMC12490786; doi:10.1126/scitranslmed.adn2601)
Supplement: Supplemental Materials, Tables, and Figures [file NIHMS2108172-supplement-Supplemental_Materials__Tables__and_Figures.pdf]

SUPPLEMENTAL MATERIALS for:

Engraftment and persistence of *HBB*-base-edited hematopoietic stem cells in nonhuman primates

Authors: Stefan Radtke*, Emily Fields, Kyle Swing, Greta Kanestrom, Jonathan S. Yen, Dnyanada Pande, Mark R Enstrom, Olivier Humbert, Mitchell J. Weiss, David R. Liu, Gregory A. Newby*, Hans-Peter Kiem,*

Corresponding authors:

S.R. (sradtke@fredhutch.org);
G.A.N. (gnewby@jhmi.edu);
H.P.K. (hkiem@fredhutch.org)

List of Supplementary Materials:

Figs. S1 to S4

Tables S1 to S5

Legend for Data file S1

Other files associated with these Supplementary Materials:

Data file S1

MDAR reproducibility checklist

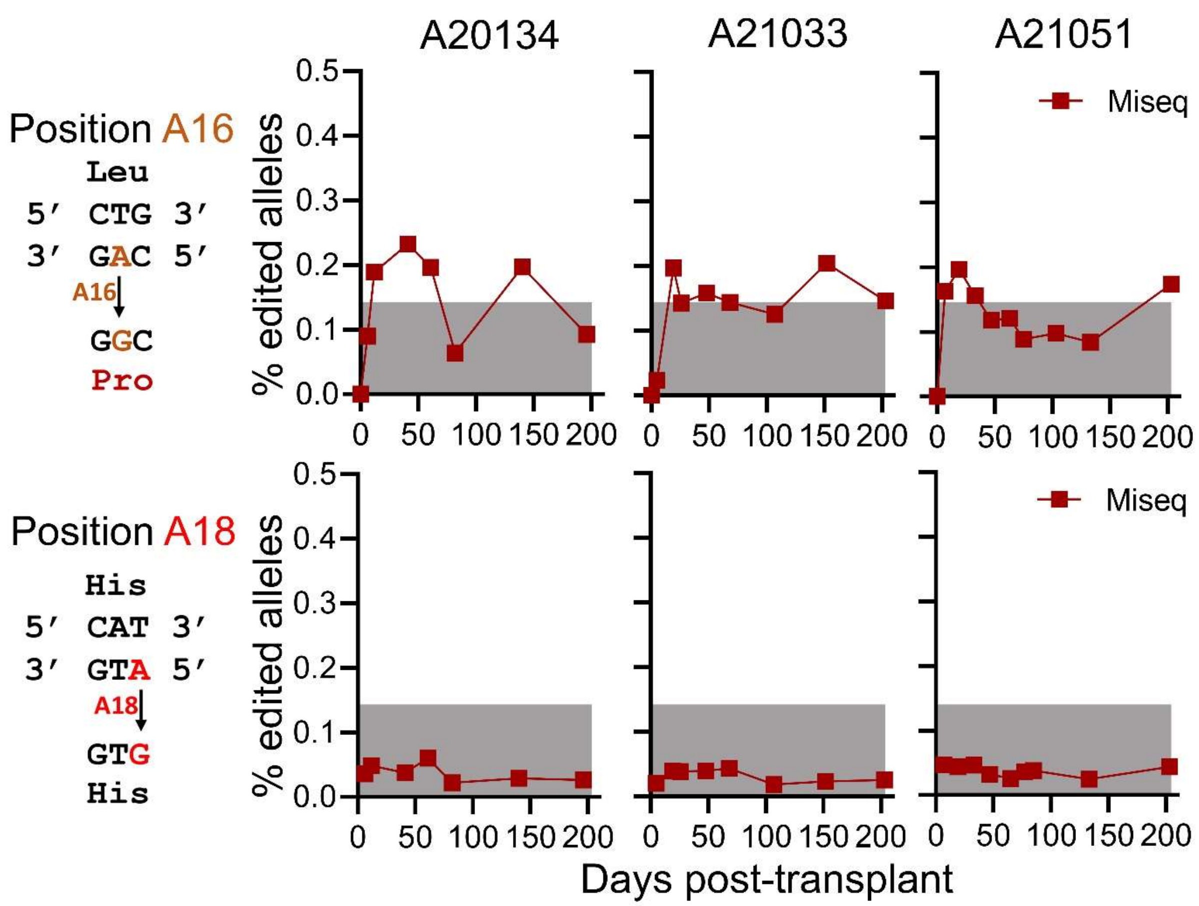

Fig. S1. Bystander editing in peripheral blood white blood cells. Longitudinal tracking of the base editing frequency at adenines A16 and A18 in PB WBCs using NGS. The gray background marks the range of background noise and was determined in unedited NGS control samples from the same animals before transplant.

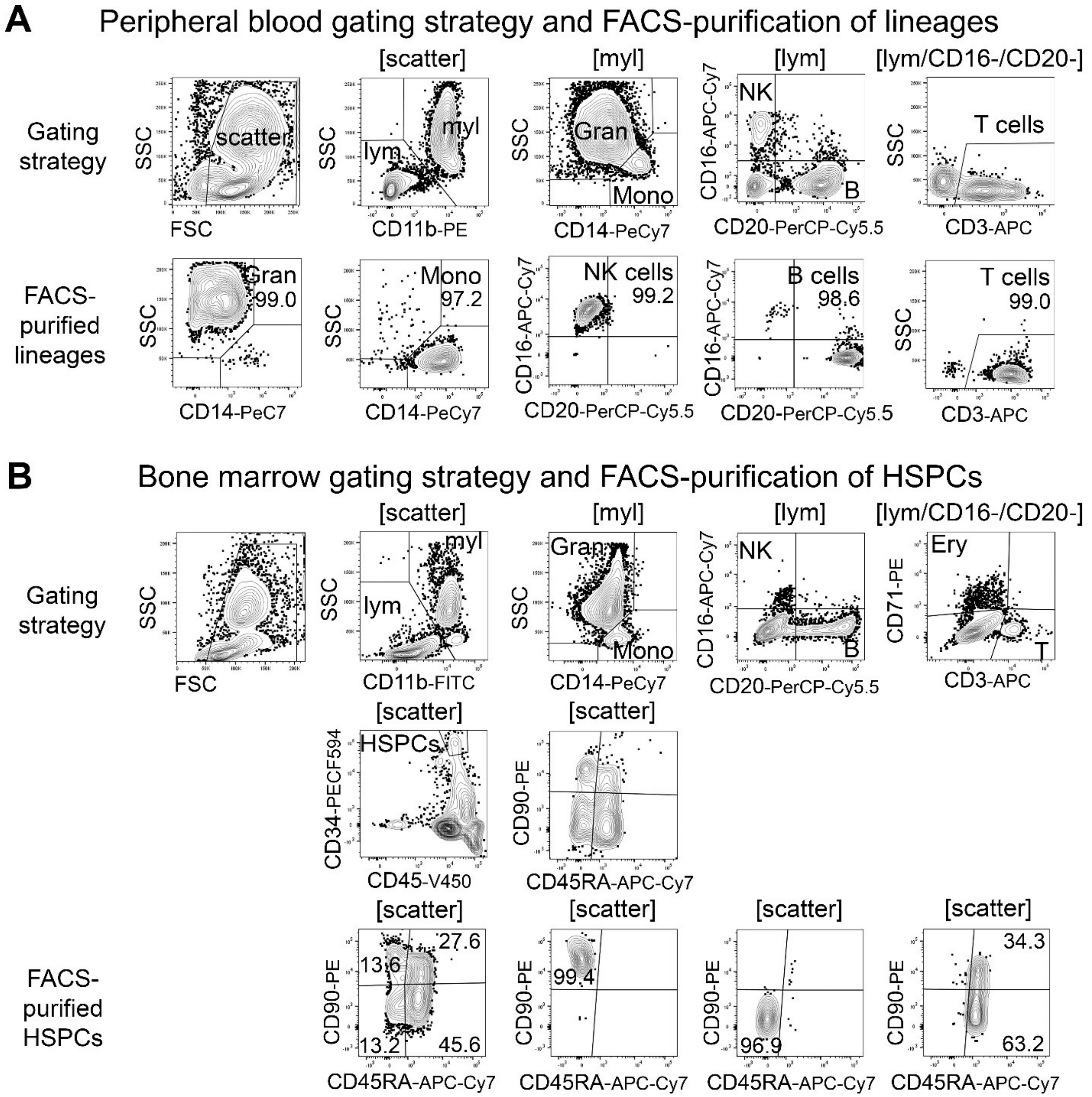

Fig. S2. Gating strategy for the analysis and FACS purification of lineages and HSPCs in the peripheral blood and bone marrow. (**A**) Gating strategy (top row) and representative quality control of FACS-purified lineages (bottom row) from the PB. (**B**) Gating strategy of lineages and HSPCs (top two rows) and representative quality control of FACS-purified HSPCs (bottom row) from the BM. Abbreviations: lym = lymphoid; myl = myeloid; Gran = granulocytes; Mono = monocytes; NK = NK cells; B = B cells; T = T cells.

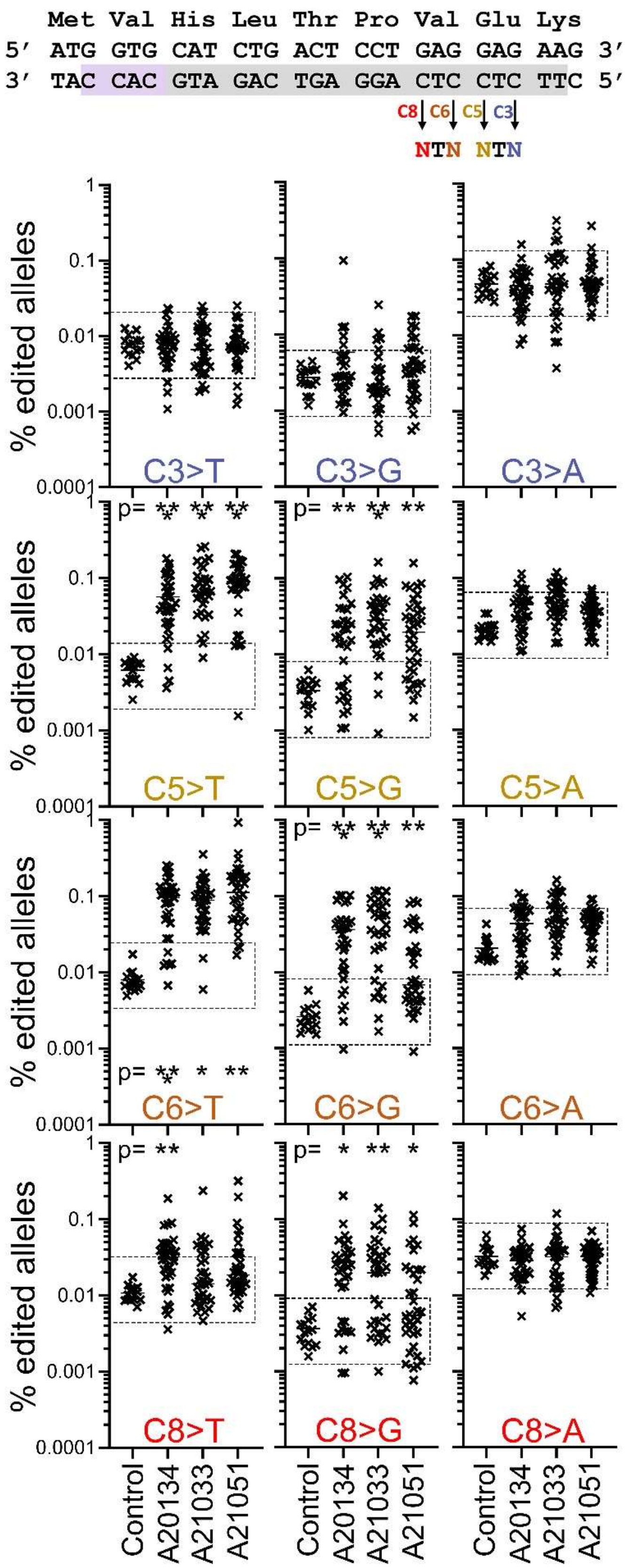

Fig. S3. Analysis of off-target cytosine editing. Top: Schematic of off-target cytosines analyzed for editing by NGS in PB and BM samples. Graphs: Frequency of substitutions in C3, C5, C6, and C8 determined by NGS in all three transplanted animals in comparison to unedited control samples (first column in each graph). Box: Range of background noise based on the unedited control samples from the same animals before transplant. Statistics: unpaired T test of data from each animal in comparison to unedited controls.

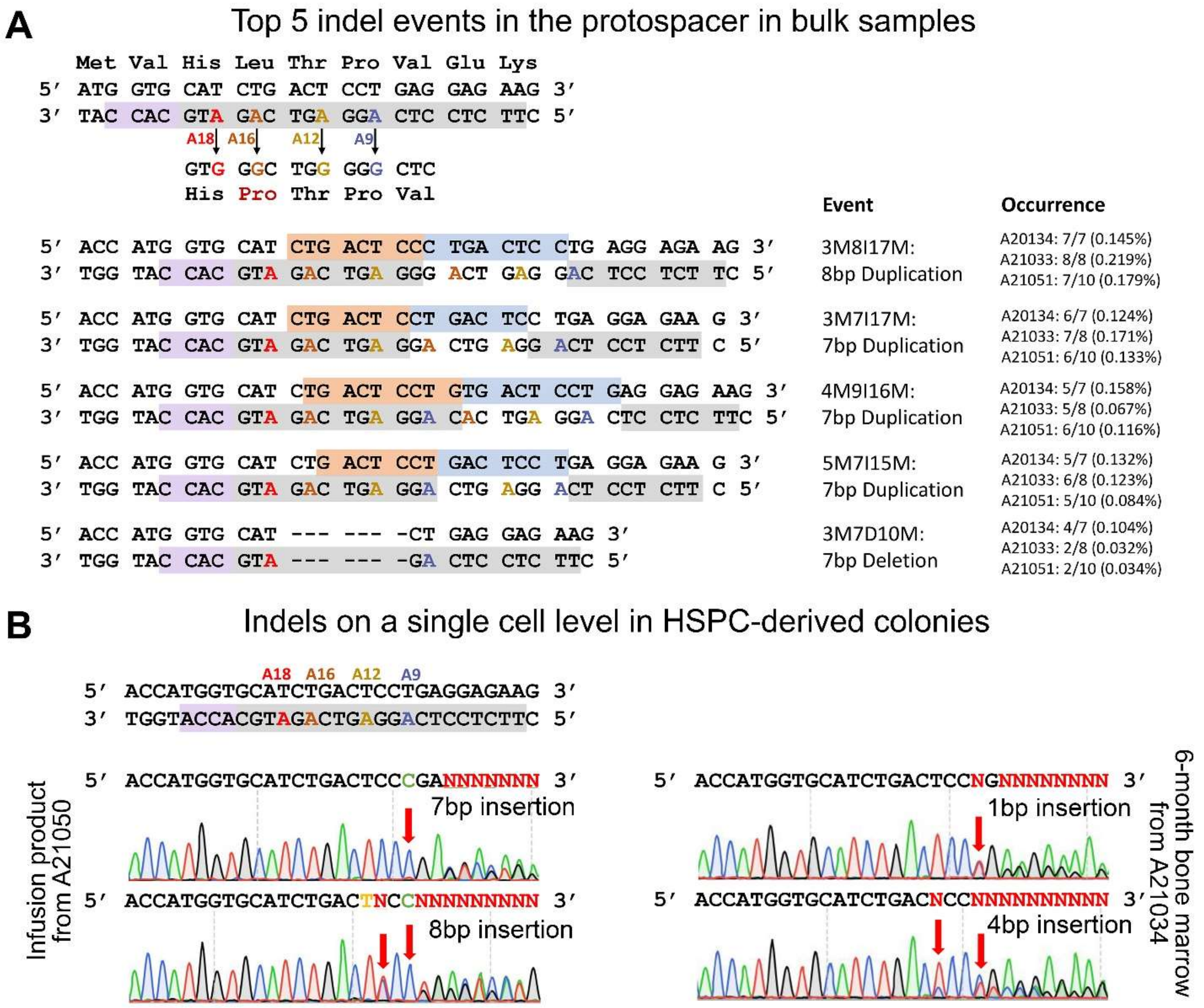

Fig. S4. Indel events in bulk samples and on a single cell level. (**A**) Top 5 indels detected by NGS in bulk samples. The number of samples and average frequency of the indel is summarized on the right. (**B**) Indels detected at the single cell level in HSPC-derived colonies (left) in the infusion product of A21050 and (right) at 6 months post-transplant in BM HSPCs from A21034, using Sanger sequencing. Determination of indels using TIDE.

Table S1. **Characteristics and parameters of the animals involved in the study.**

**Animal ID**	**A20134**	**A21033**	**A21051**
Transplant date	01-12-22	01-05-22	10-27-21
Age at transplant [yr]	1.5	3.9	2.5
**Gender**	Female	Male	Female
Weight [kg]	2.85	7.25	3.7
$CD34^+CD90^+$/kg	1.34×10^6	2.03×10^5	8.73×10^5
$CD34^+CD90^-$/kg	8.67×10^6	8.76×10^6	8.39×10^6
Days on study	203	196	201
Days to neutrophil recovery	8	8	9
Days to platelet recovery	21	22	19

Table S2. Off-target editing site details and longitudinal editing rates in peripheral blood white blood cells.

						Days post-transplant						
				**A20134**	Pre	25	33	47				
				**A21033**		13	26	40	54			
				**A21051**	Pre	12	26	40	54	68	83	118
**Site**	**Pos.**	**Edit**	**Nearby Genes**									
OT1	7	A>G	*TCEAL8* (transcription elongation factor A like 8)	**A20134**	0.239	1.425	1.085	1.005				
				**A21033**		2.926	2.166	2.060	1.661			
				**A21051**	0.229	2.898	1.751	1.168	1.023	1.055	1.212	0.774
	9	A>G		**A20134**	0.188	29.127	27.85	24.706				
				**A21033**		39.456	34.756					
				**A21051**	0.291	37.606	25.598	20.288	16.91	18.436	19.819	15.79
OT2	9	A>G	*SATB1* (SATB homeobox 1)	**A20134**	0.246	19.391	11.838	16.620				
				**A21033**		29.059	18.484					
				**A21051**	0.238	29.672	16.911	15.798	11.482	12.16	13.476	7.229
OT3	7	A>G	*CTXN2* (cortexin 2)	**A20134**	0.174	9.189	7.522	6.809				
				**A21033**								
				**A21051**	0.176	10.799	6.294	4.546	4.185	5.481	4.301	3.849
	9	A>G		**A20134**	0.316	3.350	2.764	2.677				
				**A21033**								
				**A21051**	0.189	3.538	2.147	1.890	1.451	2.083	1.638	1.519
OT4	6	A>G	*CFAP61* (cilia and flagella associated protein 61)	**A20134**	0.249	1.322	0.868	0.851				
				**A21033**		1.508	1.188	1.015	0.364			
				**A21051**	0.227	1.247	1.128	0.981	0.764	0.869	0.791	0.746
	9	A>G		**A20134**								
				**A21033**		1.557	1.359	1.215	0.858			
				**A21051**	0.212	1.766	0.982	0.749	0.629	0.738	0.787	0.538
OT5	9	A>G	*TMEM203* (transmembrane protein 203)	**A20134**	0.174	1.347	0.986	0.678				
				**A21033**		1.812	0.817	0.655	0.864			
				**A21051**	0.189	1.283	0.970	0.650	1.008	1.046	0.868	0.447
OT6	7	A>G	*TSPAN3* (tetraspanin 3)	**A20134**	0.249	1.322	0.868	0.851				
				**A21033**		1.508	1.188	1.015	0.364			
				**A21051**	0.227	1.247	1.128	0.981	0.764	0.869	0.791	0.746
OT7	5	A>G	*RPL10A* (ribosomal protein L10a)	**A20134**	0.775	1.052	1.506	1.186				
				**A21033**		1.307	1.238					
				**A21051**	0.968	0.965	1.231	1.087	0.863	1.074	1.071	1.217
OT8	9	A>G	*SOX9* (SRY-box transcription factor 9)	**A20134**	0.510	0.464	0.562	0.51				
				**A21033**		0.628	0.47	0.693	0.502			
				**A21051**	0.219	0.726	0.397	0.424	0.366	0.473	0.427	0.367

Pos.: Base position in the protospacer; OT: Off-target

Table S3. HOMER analysis of off-target editing sites.

	OT1	OT2	OT3	OT4	OT5	OT6	OT7	OT8
**Chr**	chrX	chr2	chr7	chr10	chr15	chr7	chr4	chr16
**Start**	99824931	29592101	24776690	46266763	1009834	54351558	133583443	68499297
**End**	99825173	29592295	24776882	46266998	1010067	54351784	133583678	68499485
**Strand**	+	+	+	+	+	+	+	+
**Annotation**	TTS (NM_001193726)	Intergenic	Intergenic	intron (NM_001193937, intron 18 of 26)	Intergenic	Intergenic	Intergenic	Intergenic
**Detailed Annotation**	TTS (NM_001193726)	Intergenic	LTR85c|LTR|Gypsy?	intron (NM_001193937, intron 18 of 26)	Intergenic	Intergenic	AluSg4| SINE|Alu	Intergenic
**Distance to TSS**	1568	307458	165165	171622	8017	-47992	438248	79613
**Nearest PromoterID**	NM_001193726	NM_001265648	NM_001361233	NM_001193937	NM_001261202	NM_001260729	NM_001266226	NM_001032868
**Entrez ID**	695496	696862	100428496	699814	706164	708428	718783	574208
**Nearest Refseq**	NM_001193726	NM_001265648	NM_001193386	NM_001193937	NM_001261202	NM_001260729	NM_001266226	NM_001032868
**Nearest Ensembl**	ENSMMUG 00000052429	ENSMMUG 00000019009	ENSMMUG 00000043753	ENSMMUG 00000042668	ENSMMUG 00000046202	ENSMMUG 00000010172	ENSMMUG 00000015907	ENSMMUG 00000063817
**Gene Name**	*TCEAL8*	*SATB1*	*CTXN2*	*CFAP61*	*TMEM203*	*TSPAN3*	*RPL10A*	*SOX9*
**Gene Alias**	-	-	-	C10H20orf26| C20orf26	-	-	-	-
**Gene Description**	transcription elongation factor A like 8	SATB homeobox 1	cortexin 2	cilia and flagella associated protein 61	transmembrane protein 203	tetraspanin 3	ribosomal protein L10a	SRY-box transcription factor 9
**Gene Type**	protein-coding	protein-coding	protein-coding	protein-coding	protein-coding	protein-coding	protein-coding	protein-coding

Chr: Chromosome; TSS: Transcription start site

Table S4. List of sgRNA and primers used.

Name	Vendor	Sequence
Makassar 5'-sgRNA	Synthego	5′-UUCUCCUCAGGAGUCAGAUG-3′
RhHBB535.Fmiseq (Miseq)	IDT	5’-TCGTCGGCAGCGTCAGATGTGTATAAG AGACAGACCCTGTGGAG CCACACCCTAC-3′
Miseq HBB Rhesus Long 731-752 REV 1 (Miseq)	IDT	5′-GTCTCGTGGGCTCGGAGATGTGTATAA GAGACAGCCACGTTCACCTTGCCCCACAG-3′
rhHBB3’b (Sanger)	IDT	5′-CATCAGGAGAGGACAGATCCC-3′
rhHBB5’b (Sanger)	IDT	5′-GCCAAGAGATATGTCTTAGAGG-3′

Table S5. List of antibodies used for flow cytometry and cell sorting of HSPCs.

Antigen	Fluorochrome	Clone	Brand + Catalog Number
CD34	APC	563	BD Biosciences – 562449
CD45	V450	D058-1283	BD Biosciences – 561291
CD45RA	APC-H7	5H9	BD Biosciences – 561212
CD90	PE-Cy7	5E10	BD Biosciences – 561558
CD3	BV786	SP34-2	BD Biosciences – 563918
CD4	AF700	L200	BD Biosciences – 560836
CD11b	FITC	ICRF44	Biolegend – 301330
CD14	PE-Cy7	M5E2	BD Biosciences – 557742
CD16	APC-Cy7	3GB	BD Biosciences – 557758
CD20	APC	2H7	BD Biosciences – 559776
CD45	V450	D058-1283	BD Biosciences – 561291
CD71	FITC	L01.1	BD Biosciences – 333151

Legend for Data file S1: Summary of experimental raw data illustrated in Figures 1D, 2A-D, 3A-D, 4A-C, 5A-B, 6A-D, 7A-B, S2 and S3. NGS and rhAMPseq raw data for Figures 2C-D, 4A-C, 5A-B, 7A-B and S3 were uploaded to the BioProject ID PRJNA1036686.

Data file S1. Raw data for all experiments where *n* <20.
